# Transfusion-related acute lung injury associated to intravenous immunoglobulin infusion in a pediatric patient

**DOI:** 10.31744/einstein_journal/2020RC5606

**Published:** 2020-11-25

**Authors:** José Roberto Mendes Pegler, Ana Paula Beltran Moschione Castro, Antonio Carlos Pastorino, Mayra de Barros Dorna

**Affiliations:** 1 Universidade de São Paulo Faculdade de Medicina Hospital das Clínicas São PauloSP Brazil Instituto da Criança e do Adolescente, Hospital das Clínicas, Faculdade de Medicina, Universidade de São Paulo, São Paulo, SP, Brazil.

**Keywords:** Transfusion-related acute lung injury, Immunoglobulins, Immunoglobulins, intravenous, Immunologic deficiency syndromes, Drug-related side effects and adverse reactions, Child

## Abstract

Case report of a patient with an immunodeficiency who demands regular replacement of intravenous immunoglobulin. She presented an episode of transfusion-related acute lung injury shortly after using an immunoglobulin product different than the one she usually received. The patient evolved with respiratory changes (hypoxia, dyspnea, change in pulmonary auscultation) minutes after the end of the infusion, and received non-invasive respiratory support. She was discharged after 36 hours with good outcome. The patient achieved full recovery, showing no further reactions in subsequent immunoglobulin infusions (no longer receiving the product that was used when she had the episode of transfusion-related acute lung injury). Although rare, this reaction is potentially serious and has no specific treatment other than supportive therapy. The literature is scarce regarding the risk of recurrence. The decision on whether to proceed with immunoglobulin therapy after this adverse effect should be analyzed individually, assessing the possible risks and benefits for the patient.

## INTRODUCTION

The use of intravenous immunoglobulin (IVIG) is increasing in different settings and conditions. The United States Food and Drug Administration (FDA) approved a list of products and clinical indications for the use of IVIG, and most of them are related to the prevention of infections in the context of immunodeficiency or for the treatment of an autoimmune condition.^(^^1^^)^ More than 300 inborn errors of immunity have been described,^(^^2^^)^ and many of them demand regular immunoglobulin replacement since childhood.

Adverse reactions are relatively common, occurring in 5% to 15% of the infusions, and they are mostly mild and rate-related. However, more severe reactions can occur (such as anaphylaxis, aseptic meningitis, thrombosis or acute renal failure), possibly causing disabling morbidity or mortality in rare cases.^(^^3^^)^

Transfusion-related acute lung injury (TRALI) is the leading cause of death from transfusion according to the FDA.^(^^4^^)^ It consists of an acute, noncardiogenic pulmonary edema associated with hypoxia that occurs during or within hours after a transfusion. Even though TRALI is more commonly related to red blood cells or platelets infusions, there are few cases described after IVIG administration.

In this report, we present a pediatric patient with a primary immunodeficiency and associated lung disease, who presented TRALI shortly after IVIG infusion, with a final favorable outcome.

## CASE REPORT

We report a case of a 12-year-old girl diagnosed with deficiency of cytotoxic T-lymphocyte-associated protein 4 (CTLA-4). She had a past medical history of recurrent sinusitis, otitis media, and tonsillitis since the age of three years. In the period between 7 and 9 years of age, she had recurrent episodes of autoimmune hemolytic anemia and an episode of immunological thrombocytopenia. Since the diagnosis of primary immunodeficiency (PID) with hypogammaglobulinemia at 9 years of age, she receives IVIG every 3 weeks and antimicrobial prophylaxis with sulfamethoxazole-trimethoprim.

At the age of 10 years the patient presented granulomatous-lymphocytic interstitial lung disease (GLILD), a non-infectious pulmonary complication for which she was treated with rituximab and mofetil-mycophenolate. Few months after the interruption of immunosuppressive therapy, she presented a fungal pneumonia.

In the sixth week of treatment with voriconazole for the *Aspergillus sp* lung infection, she was admitted to receive the usual dose of IVIG (580mg/kg), however, the product available (3%, Sandoglobulina^^®^^, CSL Behring, batch N° 4302500091) was differed from that one of the previous infusions. She had normal physical exam, 95% oxygen saturation (SatO^2^) in room air, and her laboratory evaluation at the same day showed normal renal function. Intravenous immunoglobulin infusion started at 1mL/kg/hour and the rate of infusion was increased every 30 minutes up to 6.7mL/kg/hour. She received a total of 600mL of the immunoglobulin solution (3% concentration), and no symptoms during the infusion were observed.

**Figure 1:**
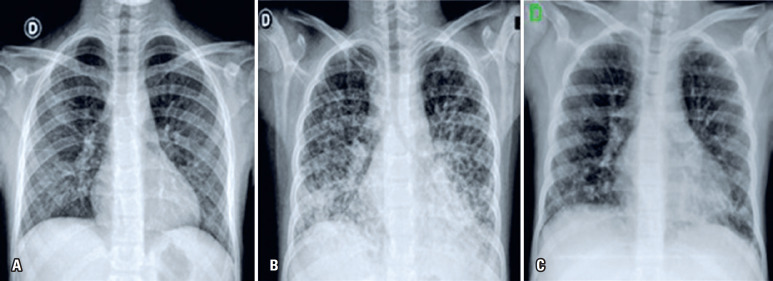
Chest X-rays of patients at baseline (A), 4 hours after intravenous immunoglobulin infusion (B), and 3 months later (C)

Half an hour after the end of the infusion, the patient complained of increasing dyspnea and presented bilateral crackles on auscultation, hypoxemia (SatO^2^ of 88% to 89% in room air), respiratory rate of 28bpm, heart rate of 125bpm, blood pressure of 114x68mmHg, and axillary temperature of 37.1°C, with no other symptoms or signs at physical examination. Non-invasive oxygen support in a fraction of inspired oxygen of 50% was offered, resulting in a SatO^2^ of 99%, and she was transferred to the emergency unit. Her chest X-ray showed signs of bilateral diffuse infiltrate, mostly on the base of the lungs, with normal cardiac silhouette ( Figure 1 ).

The patient received one single dose of intravenous furosemide (0.5mg/kg) due to the initial hypothesis of volume overload. An echocardiogram showed no abnormalities, with an ejection fraction of 79%. She had no other signs of congestion. After 24 hours the patient was with SatO^2^ >93% in room air and no other complaints, and 36 hours after the event she was asymptomatic, and was discharged.

After 3 weeks the patient received the same IVIG she was used to receive before the reaction without any adverse event. After a year of the reported adverse event, the patient is still on regular IVIG replacement without further reactions.

## DISCUSSION

Transfusion-related acute lung injury is a rare transfusion related adverse event that can be potentially severe. The management is mainly supportive, including mostly respiratory care and fluid balance maintenance. This condition is important to be recognized rapidly so the patient can be managed adequately, avoiding unnecessary interventions.

The pathophysiology of this reaction comprehends the two-hit hypothesis: the receiver neutrophils are already in an altered state due to a pathologic lung condition (first hit), and antibodies or others factors from the transfused product react against these primed neutrophils, generating damage to the vascular endothelium of the lungs (second hit).^(^^4^^)^ The patient described, due to her immunodeficiency, had a history of previous lung injury, both infectious and immune-mediated.

Although rare, there are some case reports of TRALI after IVIG in the published literature, and they were reviewed recently by Baudel et al.^(^^5^^)^ To our knowledge there are just three other cases of TRALI described in the pediatric population,^(^^5^^,^^6^^)^ and this is the first report of a child that received later doses of IVIG after experiencing TRALI.

Primary immunodeficiencies are chronic diseases and frequently demand lifelong immunoglobulin replacement therapy. In this scenario, it is important to consider that the incidence of significant adverse reaction when switching IVIG products is 15% to 18%.^(^^3^^)^ Besides, although the change from intravenous to subcutaneous infusion is an option for other adverse events of immunoglobulin,^(^^7^^)^ there are still no reports or rationale that supports this change after a TRALI episode. As a consequence, caution must be taken when considering the safety of subsequent infusions after an episode of TRALI.

In our report, the patient developed TRALI after switching to a different IVIG brand and no following reactions occurred when she received later the usually prescribed product. The published literature is limited and heterogeneous, and prevents conclusions about whether patient's clinical status, brand or specific batches of the IVIG were the most important factor determining the reaction. The risk of TRALI recurrence is not discussed in the most recently published review on the subject.^(^^5^^)^ There are three other reports of patients who received subsequent IVIG infusions after experiencing TRALI^(^^8^^-^^10^^)^ and in two of them patients also received successfully IVIG after changing the batch of the product ( Table 1 ).

**Table 1:** Characteristics of patients reported in the literature with repeated intravenous immunoglobulin infusions after transfusion-related acute lung injury

Author	Age	Gender	Diagnosis	Repeated reaction (with same or unknown batch)	Reaction when a different batch was later infused
Stoclin et al.^(^^8^^)^	57	Male	Prevention of lung transplant rejection	Yes	No
Quest et al.^(^^9^^)^	77	Female	Common variable immunodeficiency	Not performed	No
Reddy et al.^(^^10^^)^	26	Female	Myasthenia gravis	Yes *	Not performed

* This patient received intravenous immunoglobulin doses 4 days in a row, but there is no information regarding the substance lot numbers.

Due to the paucity of consistent information regarding the chances of recurrence of TRALI,^(^^11^^)^ decisions must be taken individually, considering possible risks and benefits.

This report extends the scarce data in published literature about TRALI related to IVIG, and especially regarding its recurrence. This may be helpful to others when deciding about the safety of subsequent infusions to other patients experiencing this potentially serious adverse event.
