# Supplementary material for: Transfusion-related acute lung injury associated to intravenous immunoglobulin infusion in a pediatric patient
Source: Einstein (Sao Paulo). 2020 Nov 25;18:eRC5606. doi: 10.31744/einstein_journal/2020RC5606 (PMC7690929; doi:10.31744/einstein_journal/2020RC5606)
Supplement: Translation

# Translation: Lesão pulmonar aguda relacionada à transfusão associada com infusão de imunoglobulina intravenosa em paciente pediátrico

**Authors:** José Roberto Mendes Pegler, Ana Paula Beltran Moschione Castro, Antonio Carlos Pastorino, Mayra de Barros Dorna

**Affiliations:** 1 São PauloSP Brasil Instituto da Criança e do Adolescente, Hospital das Clínicas, Faculdade de Medicina, Universidade de São Paulo, São Paulo, SP, Brasil.

**Keywords:** Lesão pulmonar aguda relacionada à transfusão, Imunoglobulinas, Imunoglobulinas intravenosas, Síndromes de imunodeficiência, Efeitos colaterais e reações adversas relacionados a medicamentos, Criança

## Abstract

Relato de caso de paciente com imunodeficiência que necessitava de reposição regular de imunoglobulina endovenosa. Ela apresentou um episódio de lesão pulmonar aguda relacionada à transfusão após uso de produto de imunoglobulina diferente daquele que recebia habitualmente. Evoluiu com alterações respiratórias (hipóxia, dispneia e alteração de ausculta pulmonar) minutos após o fim da infusão, necessitando de suporte respiratório não invasivo. A paciente recebeu alta hospitalar após 36 horas, com boa evolução. Obteve recuperação total dos sintomas, sem mais reações nas infusões subsequentes de imunoglobulina (sendo optado por não mais prescrever o produto que foi usado quando ocorreu o episódio de lesão pulmonar aguda relacionada à transfusão). Apesar de rara, essa reação é potencialmente grave, não possui tratamento específico além de terapia de suporte, e há pouca informação na literatura sobre o risco de recorrência. A decisão sobre o seguimento da terapia com imunoglobulina após esse efeito adverso deve ser analisada individualmente, avaliando os possíveis riscos e benefícios para o paciente.

## INTRODUÇÃO

O uso de imunoglubulina intravenosa (IVIG) tem aumentado em diferentes condições e situações. O órgão regulador norte-americano *Food and Drug Administration* (FDA) aprovou uma lista de produtos e indicações clínicas para o uso da IVIG, e a maioria deles está relacionada à prevenção de infecções no contexto de imunodeficiência ou para tratamento de condição autoimune.^(^^1^^)^Mais de 300 erros inatos da imunidade têm sido descritos,^(^^2^^)^e muitos deles demandam reposição regular de imunoglobulina desde a infância.

Os efeitos adversos são relativamente comuns, ocorrendo em 5% a 15% das infusões. A maioria é leve e relacionada à velocidade de infusão do produto. Porém, reações mais graves podem ocorrer (anafilaxia, meningite asséptica, trombose ou insuficiência renal aguda) e causar morbidade debilitante ou mortalidade em casos raros.^(^^3^^)^

A lesão pulmonar aguda relacionada à transfusão (TRALI - *transfusion-related acute lung injury* ) é a principal causa de morte por transfusão, de acordo com o FDA.^(^^4^^)^A TRALI consiste em edema pulmonar agudo não cardiogênico associada com hipóxia, que ocorre durante ou horas após a transfusão. Apesar disso, a TRALI é mais comumente relacionada à transfusão de hemácias ou plaquetas, e poucos casos foram descritos após infusão de IVIG.

Neste relato de caso, apresentamos uma paciente com imunodeficiência primária e doença pulmonar associada, que apresentou TRALI logo após receber infusão com IVIG, sendo o resultado favorável.

## RELATO DE CASO

Paciente do sexo feminino, 12 anos, com diagnóstico de deficiência de antígeno 4 de linfócito T citotóxico (CTLA-4 - *cytotoxic T-lymphocyte-associated protein 4* ). Tinha histórico de sinusite recorrente, otite média e amidalite desde os 3 anos de idade. Entre os 7 e os 9 anos de idade, a paciente apresentou episódios recorrentes de anemia hemolítica autoimune e episódio de trombocitopenia imune. Desde o diagnóstico de imunodeficiência primária (PID - *primary immunodeficiency* ) com hipogamaglobulinemia aos 9 anos de idade, ela recebeu infusão de IVIG a cada 3 semanas e profilaxia antimicrobiana com sulfametoxazol-trimetoprima.

Aos 10 anos de idade, a paciente apresentou doença pulmonar intersticial granulomatosa-linfocítica (GLILD - *granulomatous-lymphocytic interstitial lung disease* ), uma complicação pulmonar não infecciosa, para a qual recebeu tratamento com rituximabe e micofenolato de mofetila. Poucos meses após a interrupção da terapia imunossupressora, a paciente apresentou pneumonia fúngica.

Na sexta semana de tratamento com voriconazol para infecção pulmonar por *Aspergillus sp* , a paciente foi hospitalizada para receber dose regular de IVIG (580mg/kg), porém o produto disponível (3%, Sandoglobulina^^®^^, CSL Behring, lote 4302500091) era diferente do usado em infusões anteriores. O exame físico estava normal, com 95% de saturação de oxigênio (SatO^2^) em temperatura ambiente. Os exames laboratoriais realizados no mesmo dia mostraram função renal normal. A infusão IVIG começou em 1mL/kg/hora, e a taxa de infusão foi aumentada a cada 30 minutos, até atingir 6,7mL/kg/h. A paciente recebeu o total de 600mL da solução de imunoglobulina (concentração 3%) e permaneceu sem sintomas durante a infusão.

Cerca de 30 minutos após o fim da administração, a paciente relatou aumento da dispneia e apresentou estertores bilaterais na ausculta, hipoxemia (SatO^2^de 88% a 89% em temperatura ambiente), frequência respiratória de 28bpm, frequência cardíaca de 125bpm, pressão arterial de 114x68mmHg, temperatura axilar de 37.1°C, sem outros sintomas ou sinais. Suporte de oxigênio não invasivo em uma fração de oxigênio inspirado de 50% foi oferecido, resultando em SatO^2^de 99%, e a paciente foi transferida para unidade de emergência. A radiografia do tórax mostrou sinais de infiltrado difuso bilateral, a maioria na base dos pulmões, com silhueta cardíaca normal ( Figura 1 ).

**Figura 1:** Radiografia basal do paciente (A), 4 horas após administração de imunoglobulina intravenosa (B) e 3 meses depois (C)

A paciente recebeu dose única de furosemida endovenosa (0,5mg/kg) devido à hipótese inicial de sobrecarga de volume. O ecocardiograma não mostrou sinais de abnormalidade, com fração de ejeção de 79%. A paciente não demostrou outros sinais de congestão. Após 24 horas, ela apresentava SatO^2^>93% em ar ambiente, sem outras queixas, e, após 36 horas do evento, permanecia assintomática e teve alta hospitalar.

Após 3 semanas, a paciente recebeu infusão IVIG com o mesmo produto que utilizava anteriormente à reação sem efeitos adversos. Um ano após o relato do efeito adverso, a paciente continua em reposição regular de IVIG sem outras complicações.

## DISCUSSÃO

A lesão pulmonar aguda relacionada à transfusão é um efeito adverso raro associado à transfusão e pode ser potencialmente grave. O principal manejo é de suporte e inclui assistência respiratória e controle adequado de hidratação e balanço hídrico. É importante que o problema seja reconhecido rapidamente, para manejo adequado do paciente e, assim, evitam-se intervenções desnecessárias.

A fisiopatologia dessa reação compreende a hipótese de dois golpes: os neutrófilos do paciente que já estão em estado alterado, devido à condição pulmonar patológica (primeiro golpe), e os anticorpos ou outros fatores do produto transfundido reagem contra esses neutrófilos ativados, gerando, assim, a lesão vascular do endotélio dos pulmões (segundo golpe).^(^^4^^)^A paciente descrita, por sua imunodeficiência, tinha histórico de lesão pulmonar, tanto infecto e imunomediada.

Apesar de raros, existem, na literatura, casos relatados de TRALI após IVIG, os quais foram recentemente revisados por Baudel et al.^(^^5^^)^De acordo com nosso conhecimento, apenas três outros casos de TRALI foram descritos na população pediátrica,^(^^5^^,^^6^^)^e o presente caso é o primeiro a relatar uma criança que recebeu doses posteriores de IVIG após evento de TRALI.

Imunodeficiências primárias são doenças crônicas e frequentemente requerem terapia de reposição de imunoglobulina ao longo da vida. Portanto, é relevante considerar que a incidência de efeitos adversos significantes por ocasião da troca de produtos IVIG é de 15% a 18%.^(^^3^^)^Além disso, apesar de a troca da infusão intravenosa para a subcutânea ser uma opção para outros eventos adversos de imunoglobulina,^(^^7^^)^poucos relatos ou orientações existem para apoiar essa mudança após episódio de TRALI. Consequentemente, deve-se ter atenção ao considerar a segurança de infusões subsequentes após episódio de TRALI.

No presente relato, a paciente desenvolveu TRALI após a infusão de IVIG de uma marca diferente da usual e não apresentou reação quando, mais tarde, recebeu o produto que usava habitualmente. A literatura é limitada e heterogênea, evitando conclusões sobre o estado clínico do paciente, a marca ou os lotes específicos do IVIG serem os fatores mais importantes que determinaram a reação adversa. O risco de recorrência de TRALI não é discutido em revisão publicada recentemente.^(^^5^^)^Há outros três relatos de pacientes que receberam infusões de IVIG subsequentes após troca de lote do produto^(^^8^^-^^10^^)^( Tabela 1 ).

**Tabela 1:** Características dos pacientes relatadas na literatura que receberam infusões endovenosas de imunoglobulina repetidas após lesão pulmonar aguda relacionada à transfusão

Autor	Idade	Sexo	Diagnóstico	Reação repetida (com mesmo lote ou desconhecido)	Reação quando um lote diferente foi administrado posteriormente
Stoclin et al.^(^^8^^)^	57	Masculino	Prevenção de rejeição de transplante de pulmão	Sim	Não
Quest et al.^(^^9^^)^	77	Feminino	Imunodeficiência comum variável	Não realizada	Não
Reddy et al.^(^^10^^)^	26	Feminino	Miastenia gravis	Sim *	Não realizada

* Este paciente recebeu doses de imunoglubulina intravenosa 4 dias em uma sequência, porém não há informação relacionada ao número do lote da substância.

Devido à escassez de informações consistentes em relação às chances de recorrência de TRALI,^(^^11^^)^em tais casos, as decisões devem ser tomadas individualmente, levando em considerações possíveis riscos e benefícios.

O presente relato amplia os dados escassos existentes na literatura sobre TRALI relacionada à IVIG e especialmente em relação à sua recidiva. Nosso relato pode contribuir para a decisão sobre infusões subsequentes em pacientes que enfrentam esses eventos adversos potencialmente sérios.
